# Towards precision quantification of contamination in metagenomic sequencing experiments

**DOI:** 10.1186/s40168-019-0678-6

**Published:** 2019-04-16

**Authors:** M. S. Zinter, M. Y. Mayday, K. K. Ryckman, L. L. Jelliffe-Pawlowski, J. L. DeRisi

**Affiliations:** 1 Department of Pediatrics, Division of Critical Care, University of California, San Francisco School of Medicine, Benioff Children’s Hospital, San Francisco, CA USA; 2 Department of Epidemiology, University of Iowa, College of Public Health, Iowa City, IA USA; 3 Department of Epidemiology and Biostatistics, University of California, San Francisco School of Medicine, San Francisco, CA USA; 4 California Preterm Birth Initiative, University of California San Francisco School of Medicine, San Francisco, CA USA; 5 Department of Biochemistry and Biophysics, University of California, San Francisco School of Medicine, San Francisco, CA USA; 6 Chan Zuckerberg Biohub, San Francisco, CA USA; 7 1700 4th St, 403C, Campus Box 2542, San Francisco, CA 94158-2330 USA

**Keywords:** Metagenomics, Sequence analysis, DNA, DNA contamination, Regression analysis, Microbiota

## Abstract

Metagenomic next-generation sequencing (mNGS) experiments involving small amounts of nucleic acid input are highly susceptible to erroneous conclusions resulting from unintentional sequencing of occult contaminants, especially those derived from molecular biology reagents. Recent work suggests that, for any given microbe detected by mNGS, an inverse linear relationship between microbial sequencing reads and sample mass implicates that microbe as a contaminant. By associating sequencing read output with the mass of a spike-in control, we demonstrate that contaminant nucleic acid can be quantified in order to identify the mass contributions of each constituent. In an experiment using a high-resolution (*n* = 96) dilution series of HeLa RNA spanning 3-logs of RNA mass input, we identified a complex set of contaminants totaling 9.1 ± 2.0 attograms. Given the competition between contamination and the true microbiome in ultra-low biomass samples such as respiratory fluid, quantification of the contamination within a given batch of biological samples can be used to determine a minimum mass input below which sequencing results may be distorted. Rather than completely censoring contaminant taxa from downstream analyses, we propose here a statistical approach that allows separation of the true microbial components from the actual contribution due to contamination. We demonstrate this approach using a batch of *n* = 97 human serum samples and note that despite *E. coli* contamination throughout the dataset, we are able to identify a patient sample with significantly more *E. coli* than expected from contamination alone. Importantly, our method assumes no prior understanding of possible contaminants, does not rely on any prior collection of environmental or reagent-only sequencing samples, and does not censor potentially clinically relevant taxa, thus making it a generalized approach to any kind of metagenomic sequencing, for any purpose, clinical or otherwise.

## Main text

Metagenomic next-generation sequencing (mNGS) is a highly sensitive tool capable of detecting even single fragments of nucleic acid. While this sensitivity allows for detection of rare organisms within a much larger host background, sensitivity is a double-edged sword, as reagent and environmental contamination, ubiquitous in sequencing experiments, will also be detected and potentially misinterpreted. Contamination can be introduced by the environment, reagents, handlers, or machines at any point during the collection of the sample, the extraction of nucleic acid, or the preparation of libraries [1–6]. This can lead to results that vary widely between laboratories, reagent kits, or extraction batches [6–8], can result in false-negative or false-positive assessments [9–12], and can provide misleading information about microbiological niches [13–16]. While steps can be taken to minimize contamination, existing best practices are unable to completely prevent it or control for it; therefore, it is critical that contamination is addressed during sequencing analyses in order to prevent misleading results, particularly from low biomass samples [4, 8, 10, 12, 16–21].

In the December 2018 issue of *Microbiome*, Davis et al. present an elegant approach to the identification of contamination in metagenomic sequencing results [22]. Their approach relies on two core principles: first, that contaminant sequences are inversely correlated with total sequencing reads (the frequency-based approach), and second, that contaminant sequences are present in more controls than samples (the prevalence-based approach). Their work employs several statistical methods that culminate in a classification threshold ranging from 0 to 1. Once the threshold is set (the authors recommend 0.1 to start), a list of contaminant DNA can be compiled. Analyzing sequences according to these principles eliminates the need to assign an arbitrary threshold for removing sequences and reduces reliance on an *a priori* set list of known contaminants. Davis et al. then provide a user-friendly R package entitled *decontam* and validate their approach on multiple datasets to demonstrate robust detection of contaminating sequences in both shotgun and 16S sequencing results [22, 23].

The approach employed by Davis et al. is particularly useful in identifying contamination in low biomass samples, and the authors rightly point out that the assumptions of their approach break down when the contaminant mass (*C*) approaches the total input sample mass (*S*). For any given sample in any given mNGS experiment, the exact limit at which input sample mass becomes so small that contamination dominates the results remains unknown.

In our own work in the area of clinical mNGS, this issue has been a cause of constant concern [16, 24]. Paralleling the work of Davis et al., we sought better methods to characterize the lower limit of sample input in order to automatically both identify and quantify the contribution of each contaminating component. Here, we suggest an amendment to the method of Davis et al. This improvement relies on determining an association between sequencing read output and input mass, made possible through the incorporation of a series of precise spike-in controls. In doing so, a straightforward statistical method allows the identification and separation of contaminating components from those inherent to the sample itself, without censoring.

**Fig. 1:**
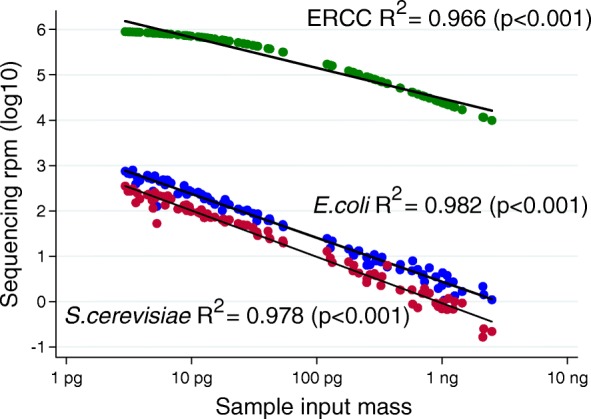
Contaminant sequencing reads are inversely proportional to sample mass. For each of *n* = 32 HeLa input masses (present in triplicate), sequencing reads for the total ERCC set (*n* = 92 different transcripts) are normalized per million (rpm) and presented in green; sequencing rpm aligning to the *E. coli* genome are presented in blue; and sequencing rpm aligning to the *S. cerevisiae* genome are presented in red. The linear regressions associating sample input mass with ERCC, *E. coli*, and *S. cerevisiae* are described with the adjusted *R*^2^ and *p* value

**Fig. 2:**
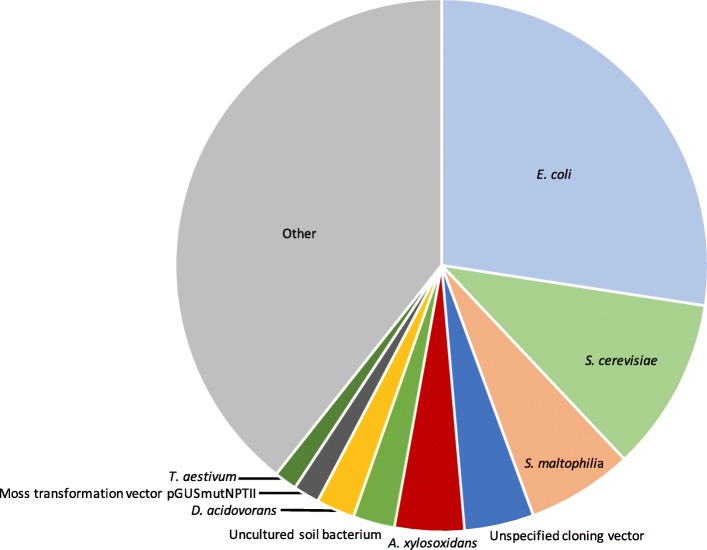
Precision quantification of microbial contamination in sequencing experiments. For each of *n* = 32 HeLa input masses (measured in triplicate), microbial contaminants were identified if the inverse linear relationship associating log_10_-transformed rpm of any given microbe with the log_10_-transformed sample mass demonstrated an adjusted *R*^2^ ≥ 0.7. By solving the equation *contaminant mass/ERCC mass = contaminant reads/ERCC reads*, the estimated mass of each contaminant in each sample was calculated. The top contaminating taxa were *E. coli* (2.59 ± 0.67 ag), *S. cerevisiae* (1.02 ± 0.30 ag), *S. maltophilia* (0.61 ± 0.49 ag), *unspecified cloning vector* (0.43 ± 0.17 ag), and *A. xylosoxidans* (0.40 ± 0.27 ag), respectively. The estimated mass of all contaminants (excluding human and low-quality reads) in each sample was 9.1 ± 2.0 ag

To demonstrate this, we prepared in triplicate a set of 32 samples consisting of between 1 picogram (pg) and 2.5 nanograms (ng) of RNA extracted from a single stock of the HeLa cell line. To each sample, we added 25 pg of a stock of 92 standardized RNA transcripts present in varying concentrations ranging from 1.4 × 10^−2^ to 3.0 × 10^−22^ mol/L (External RNA Controls Consortium, ERCC, Thermo Fisher Cat #4456740), which we have previously demonstrated can facilitate quantitation of ultra-low biomass samples [25]. Each sample then underwent library preparation (New England Biolabs Ultra II RNA Library Prep Kit) followed by 125 base paired-end sequencing on an Illumina HiSeq 4000 to a median depth of 26.9 million read-pairs per sample (interquartile range [IQR] 24.1–30.8). As depicted in Fig. 1, the log_10_-transformed sum of sequencing reads for the 92 ERCC transcripts is indeed inversely proportional to the log_10_-transformed total input sample mass (linear regression *R*^2^ = 0.966, *p* < 0.001). Using this fact, the calculation associating microbial sequencing reads and input sample mass for all microbial taxa identified in the experiment may be automated, thus directly facilitating the unbiased determination of taxa that demonstrate contaminant behavior. Simply by solving the equation *contaminant mass/ERCC mass = contaminant reads/ERCC reads*, the mass contribution of each contaminant may be quantified for each experiment (Fig. 2). In this set of samples, the sum mass of all contaminants was 9.1 ± 2.0 attograms, which suggests that samples of less than 10 ag may be overwhelmed by contamination bias and thus would be unusable. This measurement incorporates only high-quality microbial reads and could be adapted to include other contaminating reads such as human-derived or low-quality microbial reads as needed. Of note, statistical confidence in the ability to estimate the molar contribution of each contaminating taxa actually increases when the experimental batch contains samples that vary over a wide range of input masses, suggesting that sub-sampling input nucleic acid across a batch of samples to approximately the same input mass prior to library preparation may, in fact, be contraindicated.

After microbial taxa are binned as either contaminant or true constituent of the microbiome, Davis et al. propose that contaminant taxa are censored from the dataset and nicely demonstrate a reduction in batch effect and other experimental improvements. However, as described by the authors, one significant limitation of the approach is that “*decontam* assumes that contaminants and true community members are distinct from one another.” In our view, such binary assignments are not realistic for a number of important microbes in numerous experimental situations. Consider the example of a human patient harboring an *Escherichia coli* bloodstream infection. As *E. coli* appears to be a ubiquitous laboratory contaminant, attempts to sequence the metagenome from a blood sample would produce a final *E. coli* sequencing count with contributions from both reagent contamination and the true microbiome. Disregarding *E. coli* as a component of the microbiome based on its identification as a contaminant would result in a false negative report, which could be disastrous in the field of clinical metagenomics for infectious disease diagnostic purposes. Similar vignettes can be described for numerous microbes that are both pathogenic and common laboratory contaminants, including *Staphylococcus aureus* and *Pseudomonas aeruginosa*.

**Fig. 3:**
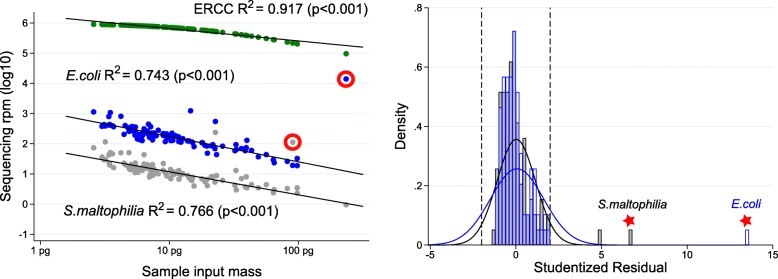
Identification of outliers among contaminant microbes. Left: for each of *n* = 97 serum sample RNA input masses, sequencing reads for the total ERCC set (*n* = 92 different transcripts) are normalized per million (rpm) and presented in green; sequencing rpm aligning to the *E. coli* genome are presented in blue; and sequencing rpm aligning to the *S. maltophilia* genome are presented in grey. The linear regressions associating sample input mass with ERCC, *E. coli*, and *S. cerevisiae* are described with the adjusted *R*^2^ and *p* value. Right: a histogram of the studentized residual for each observation informing the linear regression between log_10_-transformed sequencing reads (*E. coli* in blue, *S. maltophilia* in grey) and log_10_-transformed sample input mass. Studentized residuals approximate a near-normal distribution between − 2 and + 2 such that outliers can be rapidly identified (red)

We propose the following logical extension of Davis et al. After the establishment of the inverse linear relationship between contaminant reads and input sample mass in an experiment, the quantity of microbial reads for a given taxa in a given sample can be described according to its deviation from the value predicted by the above-described linear regression. Dividing this value by the standard error produces the studentized residual, which can serve to identify outliers to the linear relationship while also accounting for varying statistical power at different points along the linear regression [26]. To demonstrate the value of this approach, we prepared sequencing libraries from 97 de-identified serum samples from pregnant women collected with informed consent (UCSF IRB 12-090702). As described above, RNA was extracted from 100 microliters (uL) of serum, and then combined with 25 pg ERCC RNA spike-in control set. Following library preparation, as described in [23], 125 base paired-end sequencing on an Illumina NovaSeq instrument was conducted to a median depth of 34.6 million read-pairs per sample (IQR 28.3–43.6). Using the above-described workflow, we identified numerous contaminant microbes including both non-pathogenic laboratory contaminants such as *Delftia acidovorans* and *Achromobacter xylosoxidans* as well as potentially pathogenic organisms including *Escherichia coli* and *Stenotrophomonas maltophilia*. Rather than censoring *E. coli* results from the dataset, we use the studentized residual approach to identify a patient with *more E. coli than expected from contamination alone* (Fig. 3). Phylogenetic tree analysis suggests the *E. coli* in this sample to be phylogenetically distinct from the *E. coli* in our no-template controls. Using this methodology, we noted an additional patient sample as possessing a quantity of *S. maltophilia* in excess of what was expected from contamination alone. Orthogonal confirmation of the presence of *E. coli* and *S. maltophilia* RNA in the original sera was performed using custom reverse transcription primers followed by Sanger sequencing^1^. Of note, to avoid the potential for confusing distinct organisms with highly similar genomes, we recommend examining each taxa at the highest resolution (lowest phylogenetic level) supported by the depth of sequencing and the detection within the metagenome. The non-human (microbial) reads from this dataset are available under the Sequence Read Archive (SRA) BioProject ID PRJNA516238.

In summary, Davis et al. present an intuitive and straightforward approach to identifying contamination in metagenomic sequencing experiments. When microbe sequencing quantity is inversely proportional to total sample input mass, it is suspicious for contamination; we thus suggest that assessing the studentized residual for each sample can provide a probabilistic assessment of the degree to which a contaminant might also be present in the true sample metagenome. The inclusion of ERCC controls provides the additional benefit of allowing sample input mass to be calculated even for picogram-level samples. In short, this statistical approach allows an investigator to separate the estimated contribution from contamination from the true sample-derived component without censoring the organism from all further analyses. Importantly, our method assumes no prior understanding of possible contaminants and does not rely on any prior collection of environmental or reagent-only sequencing samples, thus making it a generalized approach to any kind of metagenomic sequencing, for any purpose, clinical or otherwise.

## Abbreviations

ERCC: External RNA Controls Consortium
IQR: Interquartile range
mNGS: Metagenomic next-generation sequencing
SRA: Sequence Read Archive

## Footnotes

1 The presence of *E. coli* and *S. maltophilia* RNA in the original sera was confirmed using custom PCR primers for the following inserts with 100% BLAST homology for their respective species. *E. coli* insert: TCAGCACGATTTCAGTCTGAGTCGGACATTCAGCAGTGATACCCGCAGGCAGCTGATGGTCAACAGGATGAGAGAAACCCAGAGACAGGTTAATCACATTGCCTTTAACCGCTGCACGGTAACCTACACCAACCAGCTGCAGCTTCTTAGTGAAGCCTTCGGTAACACCGATAACCATTGAGTTCAGCAGGGCACGCGCGGTACCAGCCTGTGCCCAACCGTCTGCGTAACCATCACGCGGACCGAAGGTCAGGGTATTATCTGCATGTTTAACTTCAACAGCATCGTT. *S. maltophilia* insert: ATAGCCCTGTATCTGAAAGGGCCATTTCAGTGAAGACGAGTAGGGCGGGGCACGTGAAACCCTGTCTGAACATGGGGGGACCATCCTCCAAGGCTAAATACTACTGACCGACCGATAGTGAACCAGTACCGTGAGGGAAAGGCGAAAAGAACCCCGGAGAGGGGAGTGAAATAGAACCTGAAACCGTGTGCGTACAAGCAGTAGGAGCTCCGCAAGGAGTGACTGCGTACCTTTTGTATAATGGGTCAGCGACTTACTG
